# The effects of Thalamic Deep Brain Stimulation on speech dynamics in patients with Essential Tremor: An articulographic study

**DOI:** 10.1371/journal.pone.0191359

**Published:** 2018-01-23

**Authors:** Doris Mücke, Anne Hermes, Timo B. Roettger, Johannes Becker, Henrik Niemann, Till A. Dembek, Lars Timmermann, Veerle Visser-Vandewalle, Gereon R. Fink, Martine Grice, Michael T. Barbe

**Affiliations:** 1 IfL–Phonetics, University of Cologne, Cologne, Germany; 2 University Hospital Cologne, Department of Neurology, Cologne, Germany; 3 University Hospital Marburg, Department of Neurology, Marburg, Germany; 4 University Hospital Cologne, Department of Stereotaxy and Functional Neurosurgery, Cologne, Germany; University of Pennsylvania Perelman School of Medicine, UNITED STATES

## Abstract

Acoustic studies have revealed that patients with Essential Tremor treated with thalamic Deep Brain Stimulation (DBS) may suffer from speech deterioration in terms of imprecise oral articulation and reduced voicing control. Based on the acoustic signal one cannot infer, however, whether this deterioration is due to a general slowing down of the speech motor system (e.g., a target undershoot of a desired articulatory goal resulting from being too slow) or disturbed coordination (e.g., a target undershoot caused by problems with the relative phasing of articulatory movements). To elucidate this issue further, we here investigated both acoustics and articulatory patterns of the labial and lingual system using Electromagnetic Articulography (EMA) in twelve Essential Tremor patients treated with thalamic DBS and twelve age- and sex-matched controls. By comparing patients with activated (DBS-ON) and inactivated stimulation (DBS-OFF) with control speakers, we show that critical changes in speech dynamics occur on two levels: With inactivated stimulation (DBS-OFF), patients showed coordination problems of the labial and lingual system in terms of articulatory imprecision and slowness. These effects of articulatory discoordination worsened under activated stimulation, accompanied by an additional overall slowing down of the speech motor system. This leads to a poor performance of syllables on the acoustic surface, reflecting an aggravation either of pre-existing cerebellar deficits and/or the affection of the upper motor fibers of the internal capsule.

## Introduction

We here investigated articulatory parameters of speech motor control, specifically of the labial and lingual systems in Essential Tremor (ET) patients treated with Deep Brain Stimulation (DBS) of the nucleus ventralis intermedius (VIM-DBS) of the thalamus. ET is the most common adult movement disorder with an estimated prevalence of about 0.9% [1]. Clinically, ET presents with bilateral, postural, or kinetic tremor of hands and forearms, sometimes also of legs, trunk, head, and voice [2]. This may cause significant disability, interfere with activities of daily living, and reduce quality of life. For medication refractory cases, DBS of the ventral intermediate nucleus offers an established, effective and safe treatment option [3].

Clinical studies demonstrated that dysarthria is one of the most common side-effects of VIM-DBS in ET [4–6]. Therefore, despite tremor improvement, VIM-DBS can have deleterious effects on speech leading to reduced voicing and imprecise oral articulation during the production of consonants and vowels. So far, parameters in the acoustic dimension related to the overall speaking rate and the syllable internal coordination have been used to quantify stimulation induced dysarthria in ET. For overall speaking rates, [7] found that ET patients with additional cerebellar signs (such as functionally incapacitating intention tremor or atactic gait) exhibited increased syllable duration compared to ET patients without cerebellar signs and that thalamic DBS had no effect on the speaking rate.

However, the picture changes when looking at articulatory patterns on the subsyllabic level. Acoustic studies with patients suffering from ET [8] or multiple sclerosis [9] showed that during fast syllable repetition tasks, where subjects repeat sequences such as /pa/, /ta/, or /ka/, VIM-DBS leads to a deterioration of glottal and oral control, measured as a decrease in voiceless intervals and an increase in spirantization of stop consonants on the acoustic surface, respectively. For patients suffering from Parkinson’s disease (PD), imprecise oral articulation under DBS treatment is also reported. More specifically, the stimulation of the subthalamic nucleus (STN-DBS) [10–14] and the caudal zona incerta (cZi-DBS) [13,14] lead to an increase of spirantization, but variation within the groups were rather high for STN-DBS compared to cZi-DBS [13,14]. High speaker-specific variation for STN-DBS in PD-patients was also reported by [10]. In an articulatory study using electropalatography (EPG), they recorded two PD patients by using individual artificial palates with incorporated touch-sensitive electrodes to capture contacts of the tongue with the palate during speech, but the expected target undershoot in the EPG contact profiles was only found for one of the two PD patients. However, EPG studies do not provide information about the activation interval of a movement including duration, velocity profiles and displacements since they are restricted to full tongue-palate contacts during consonantal targets.

Based on previous studies, we can assume that DBS in the VIM can lead to a decrease in articulatory precision during the production of stop consonants in ET patients. However, it is not possible to determine whether the articulatory target undershoot (signaled by the leaking stop closures) is due to a general slowing down of the speech motor system in terms of decelerated velocity profiles of the primary constrictors lips and tongue or disturbed articulation (e.g., a target undershoot caused by problems with relative phasing of articulatory movements). To shed light on the question how the underlying articulatory movement patterns are coordinated in ET patients with activated and inactivated stimulation, we directly observe the lip and tongue movements measured in the articulatory dimension. Therefore, we combine acoustic and articulatory measures to predict the nature of the articulatory deficits leading to a deterioration in speech on the acoustic outcome. We use Electromagnetic Articulography (EMA). To our knowledge EMA and DBS in general were not used in combination so far. Based on previous reports on stimulation induced dysarthria in ET, we had the following hypotheses for the comparison between controls and ET patients in DBS-OFF condition and for the comparison between ET patients in DBS-OFF and in DBS-ON condition:

1. In the acoustic waveform, we expected to find longer syllable durations, higher amounts of frication, and less voiceless intervals (control < DBS-OFF < DBS-ON).
2. In the articulographic data, we expected to find an increase of articulatory miscoordination in terms of articulatory undershoot of desired motor goals resulting in frication, accompanied by an overall slowing-down of labial and lingual speech gestures (control < DBS-OFF < DBS-ON).

## Method

### Participants

We recorded 12 ET patients (8 males, 4 females) with *activated* stimulation (DBS-ON) and *inactivated* stimulation (DBS-OFF) aged between 31 and 73 years (mean 62 years old, SD = 12) and 12 age- and gender-matched healthy control speakers (mean 61 years old, SD = 12). All speakers were right-handed and native speakers of Standard German.

ET patients had VIM-DBS surgery at least 4 months prior to participation in the study (see Table 1). The occurrence of postoperative stimulation-induced dysarthria was a criterion for inclusion (according to clinical observations), and voice tremor was not an exclusion criterion (one patient suffered from voice tremor under deactivated stimulation which dissolved by switching on DBS). The order of stimulation (DBS-ON and DBS-OFF) states was randomized to avoid bias of practice effects or fatigue. Before each testing, the stimulation settings were maintained for at least 20 minutes. The patients’ regular stimulation parameters were used during the DBS-ON condition (S1 Appendix). Tremor severity during DBS-ON and DBS-OFF was assessed using the Fahn-Tolosa-Marin Tremor Rating Scale, part A and B (TRS; [15]). The TRS is the standard scale to quantify Tremor in ET. It is divided into three parts (A, B and C). Part A describes tremor severity at rest, with posture holding and action/intention maneuvers for nine parts of the body. Part B describes tremor severity at writing, drawing, and pouring water. Part C assesses functional disability. Additionally, patients and controls had to rate their subjective “ability to speak” on a Visual Analogue Scale (VAS, ranging from 0 cm–‘normal’–to 10 cm–‘worst’ in 1mm increments). Clinical voice impairment was measured with the Voice Handicap Index (VHI; [16]). The VHI is a questionnaire measuring overall voice impairment in everyday life [17] and therefore not suitable for DBS-ON and DBS-OFF comparisons. Accordingly, the VHI was completed once by the patients at the day of testing. The patients were asked to rate their speech condition in the timespan of the last four weeks. Despite not being intended to detect articulatory changes, we present the VHI data on a descriptive level to give an overall idea about the speech impairment in our patients.

**Table 1:** Characteristics of 12 ET patients and 12 control speakers.

	Patients				Controls	
No.	Sex	Age	Years of disease duration	Months of VIM-DBS	Sex	Age
1	m	66	22	25	m	63
2	m	70	29	5	m	69
3	m	31	9	105	m	30
4	m	73	34	56	m	73
5	f	53	3	47	f	52
6	f	59	4	33	f	58
7	m	54	7	67	m	54
8	f	60	14	93	f	56
9	f	61	39	4	f	68
10	m	67	24	66	m	63
11	m	72	9	7	m	70
12	m	73	7	81	m	75
**MEAN** **(±SD)**		**61.58** **(±11.93)**	**16.75** **(±12.40)**	**49.08** **(±34.8)**		**60.92** **(±12.31)**

### Implantation/electrode localization

**Fig 1:**
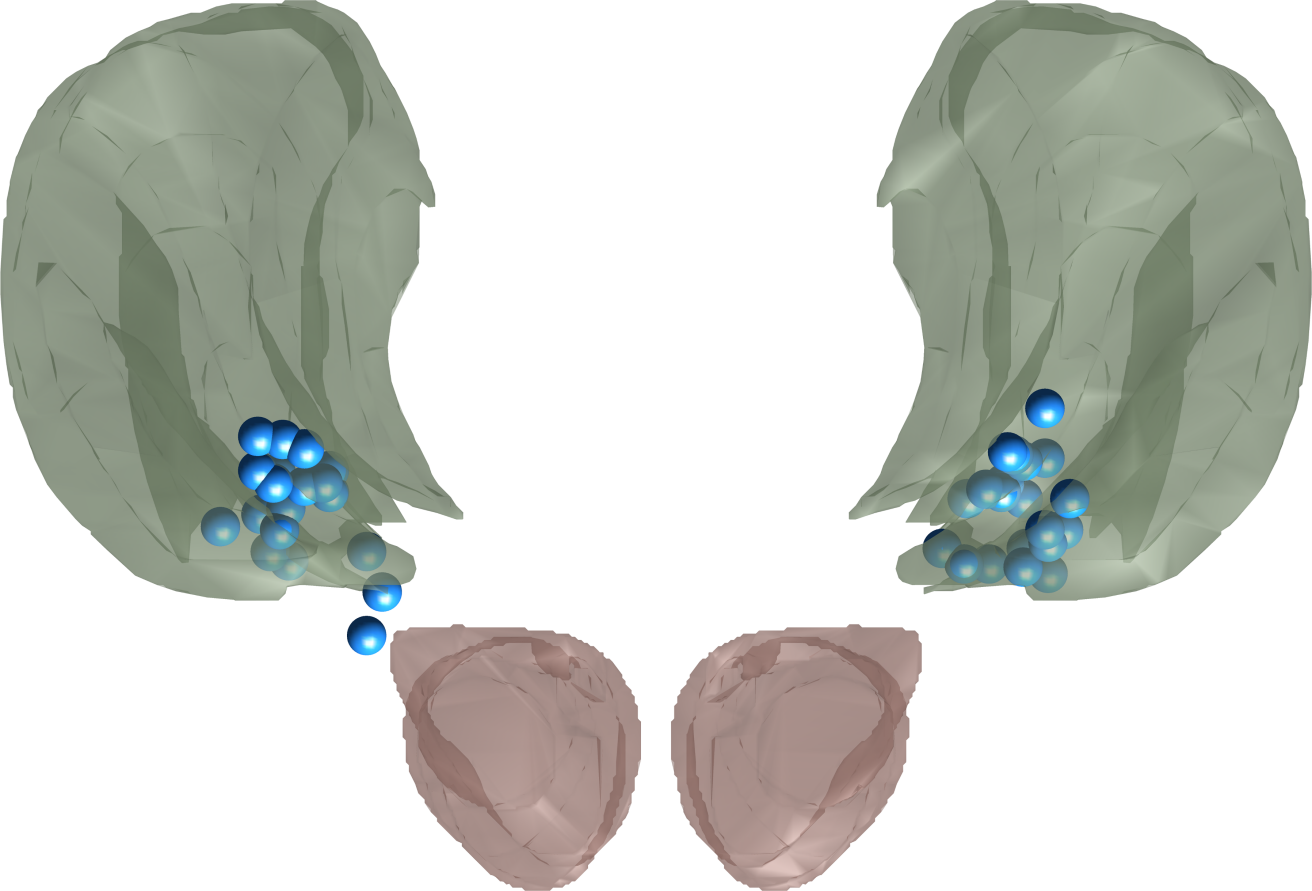
Projection of all active contacts (blue) onto a neuroanatomical atlas [19]. Green: VIM, red: red nucleus. Note that most active contacts lay inside the VIM proper or at its ventral border while two contacts lay slightly more ventral in the zona incerta.

DBS implantations were conducted as described in [18]. Surgical planning targeted the ventral border of the VIM, but depending on intraoperative test stimulation, electrodes were sometimes implanted slightly more ventral with one contact in the Zona incerta [18]. Electrode locations were confirmed via either intraoperative stereotactic X-ray after fixation or postoperative cranial CT scans, which were coregistered to preoperative MRI imaging. Electrode locations were then standardized into stereotactic brain space [18]. Stereotactic coordinates of the active electrode contacts are listed in S1 Appendix, and a visualization of the active contacts with surrounding atlas anatomy [19] can be seen in Fig 1. The mean location of the most ventral contacts was 10.7 mm lateral, 6.3 mm posterior and 1.3 mm ventral to the mid-commissural point while the mean location of active electrodes was 11.6 mm lateral, 4.2 mm posterior and 0.6 mm dorsal to the mid-commissural point.

### Recordings

**Fig 2:**
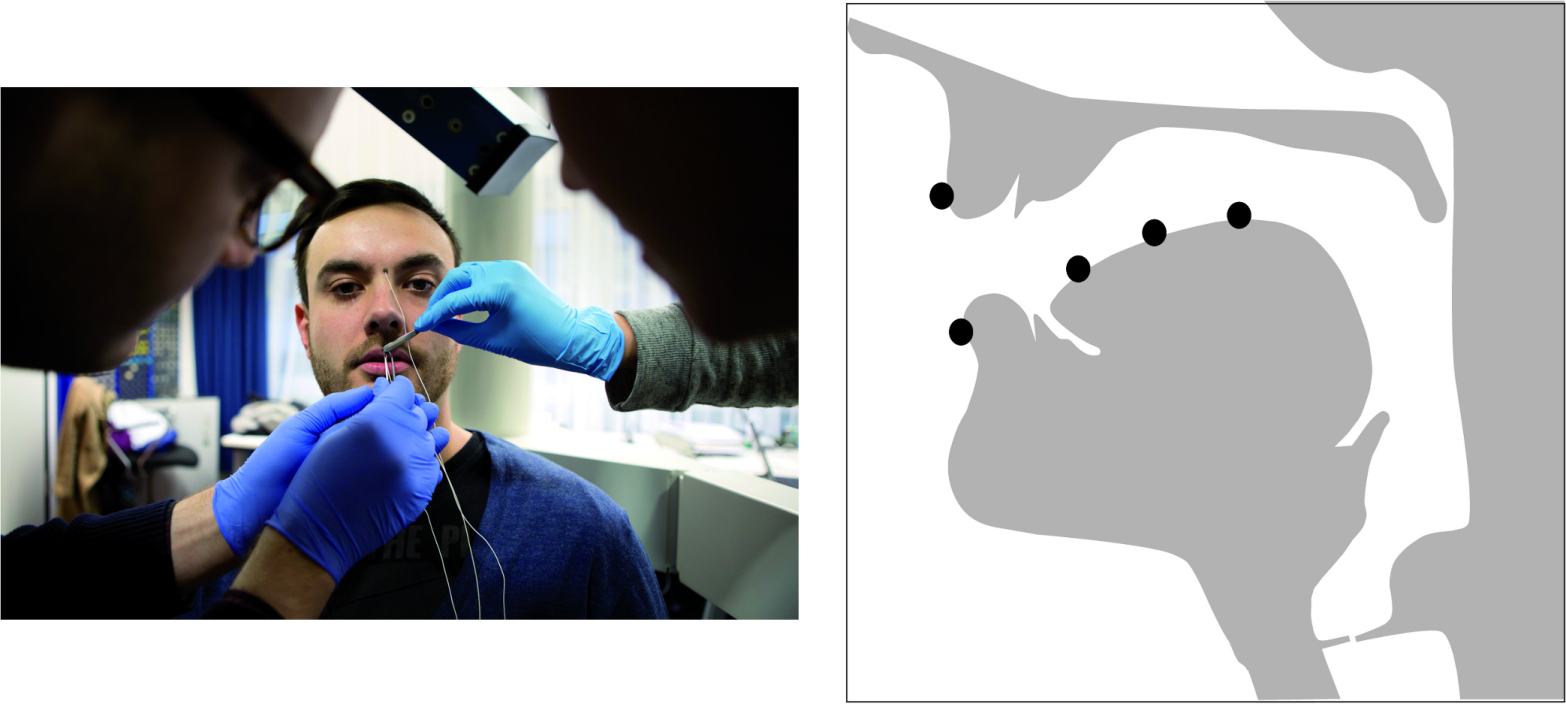
Articulograph (left) and location of sensors in the sagittal plane (right). The person on the left side on the picture (by Fabian Stürtz) is neither a patient nor a subject of the study.

The articulatory data were recorded with a 3-dimensional articulograph (Carstens Medizinelektronik; AG501) at the IfL–Phonetics lab at the University of Cologne (see Fig 2). To track the movements of the articulators, we placed sensors on the upper and lower lip, tongue tip, tongue blade, and tongue dorsum. The sensors remained at the articulators for both measurements (DBS-ON and DBS-OFF) to guarantee comparability of the data. The acoustic data (time-synchronized) were recorded using a condenser microphone (AKG C420 headset) sampled at 48kHz, 16bit.

In order to push the speech motor system to its limits, we used fast syllable repetition tasks (diadochokinesis, DDK) featuring CV syllables involving different places of articulation (POA): one labial set (i.e., /pa/ (lips) and two lingual sets (i.e., /ta/ (alveolar, tongue tip) and /ka/ (velar, tongue dorsum)). Patients were instructed to produce the syllables as fast as possible on one single breath. For the DDK analysis, we used 10 syllable cycles from each /pa/, /ta/, /ka/ production. As in [8], we discarded the first three syllable cycles to avoid effects of prosodic boundaries [20]. In total, 2160 tokens went into the statistical analysis, 10 syllable cycles x 2 repetitions x 3 POA x 3 groups (12 healthy age- and gender-matched control speakers and 12 ET patients in DBS-OFF and DBS-ON condition).

### Labelling procedure/ variables

All acoustic data were displayed and labelled by hand in Praat [21]. Annotations were carried out by using the speech waveform and a wide-band spectrogram.

The labelling procedure included the visually inspection of the acoustic waveform and the spectrogram. We used the following annotation criteria: The duration of the vowel portion for the measure “voicing-to-syllable ratio” was defined from a substantial increase to a drop in of the second formant in the spectrogram. The voiced portions during the consonantal closure for the measures “voicing-to syllable ratio” and “voicing-during-closure” were identified as frequency periodic structure above 500 Hz characterized by vertical and/or horizontal striations along the spectrogram, where voicing continuous into the constriction phase as a result of ongoing vocal-fold vibrations. For the “frication-during-closure” measure, turbulent noise during the consonantal closure was identified in terms of non-transient, aperiodic energy at a high frequency range in the spectrogram (leaking closures lead to the aerodynamic consequence of turbulence in the partially blocked airflow). Note that in contrast to [14], the measures frication-during-closure and voicing-during-closure are binary categorizations and not temporal measures. We computed the following variables in line with the label criteria reported in [8].

(1) **Syllable duration** (ms): Duration of the entire syllable cycle from the onset of the consonantal constriction to the offset of the following vowel (i.e. substantial decrease of the amplitudes of the second vowel formant).
(2) **Voicing-to-syllable ratio**: Duration of voiced portions relative to the duration of the entire syllable cycle, including vowel duration and potentially voiced portions of the consonant.
(3) **Voicing-during-closure**: Binary categorization of constriction exhibiting voicing or not. If voicing energy lasted longer than 20 ms during the closure (and therefore cannot be attributed to coarticulation of the preceding vowel), we counted the token as having voicing-during-closure (adapted from [22]).
(4) **Frication-during-closure**: Binary categorization of constriction exhibiting frication or not. If there was aperiodic energy/turbulent noise during consonant production, we counted the token as having frication-during-closure (adapted from [22]). No threshold is used here, since frication is caused by imprecise articulation in the oral tract and cannot be attributed to coarticulation.

In the articulatory domain, we identified gestural landmarks for the consonantal gestures. All kinematic data were labelled within the EMU speech database system [23].

**Fig 3:**
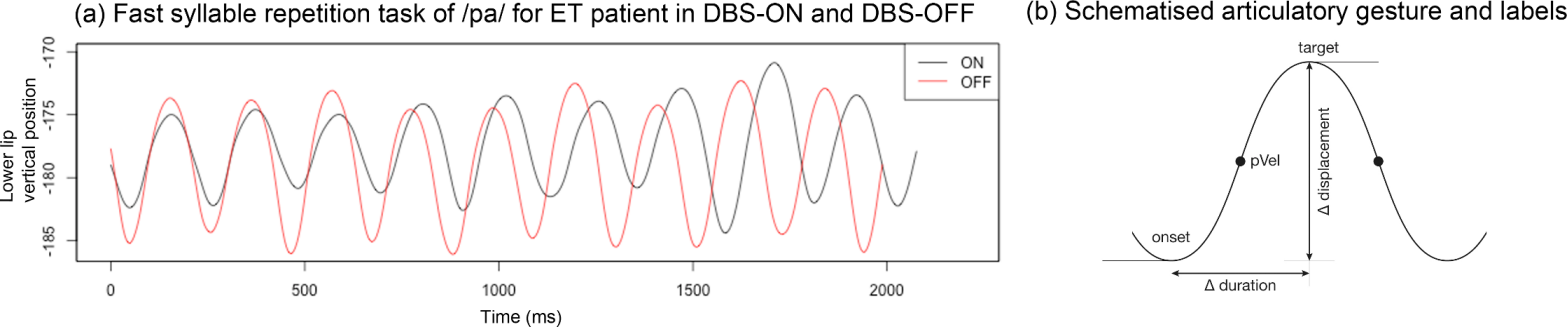
(a) Lower lip trajectory for fast syllable repetition task /pa/ for ET patient (black = DBS-ON; red = DBS-OFF;) and (b) schematized articulatory gesture with relevant landmarks.

Kinematic annotation for the consonantal gestures /p, t, k/ was performed with respect to the articulators involved (lower lip vertical position for /p/, tongue tip vertical position for /t/, tongue dorsum vertical position for /k/). We labelled the onset (start), peak velocity, and maximum target of the consonantal gestures using zero-crossings in the respective velocity and acceleration traces. Fig 3 displays (a) averaged trajectories for 10 syllable cycles of /pa/ for an ET patient with DBS-ON (black lines) and DBS-OFF (red lines) and (b) a schematized, simplified gesture illustrating the relevant landmarks (e.g., onset, peak velocity, target).

This labelling procedure allowed us to compute the following variables employed in a mass-spring model [24]:

(5) **Duration of acceleration phase** (ms): Time interval from the onset to the peak velocity of the consonantal gesture.
(6) **Duration of deceleration phase** (ms): Time interval from peak velocity to the target of the consonantal gesture.
(7) **Displacement** (mm): Amplitude (vertical) ranging from the onset to the target of the consonantal gesture.
(8) **Peak velocity** (mm/s): Maximum velocity of the gestural movement identified at a/the zero-crossing in the acceleration trace.
(9) **Stiffness** (pvel/displacement): Temporal-spatial parameter that relates the peak velocity (8) to the maximum displacement (7).

**Fig 4:**
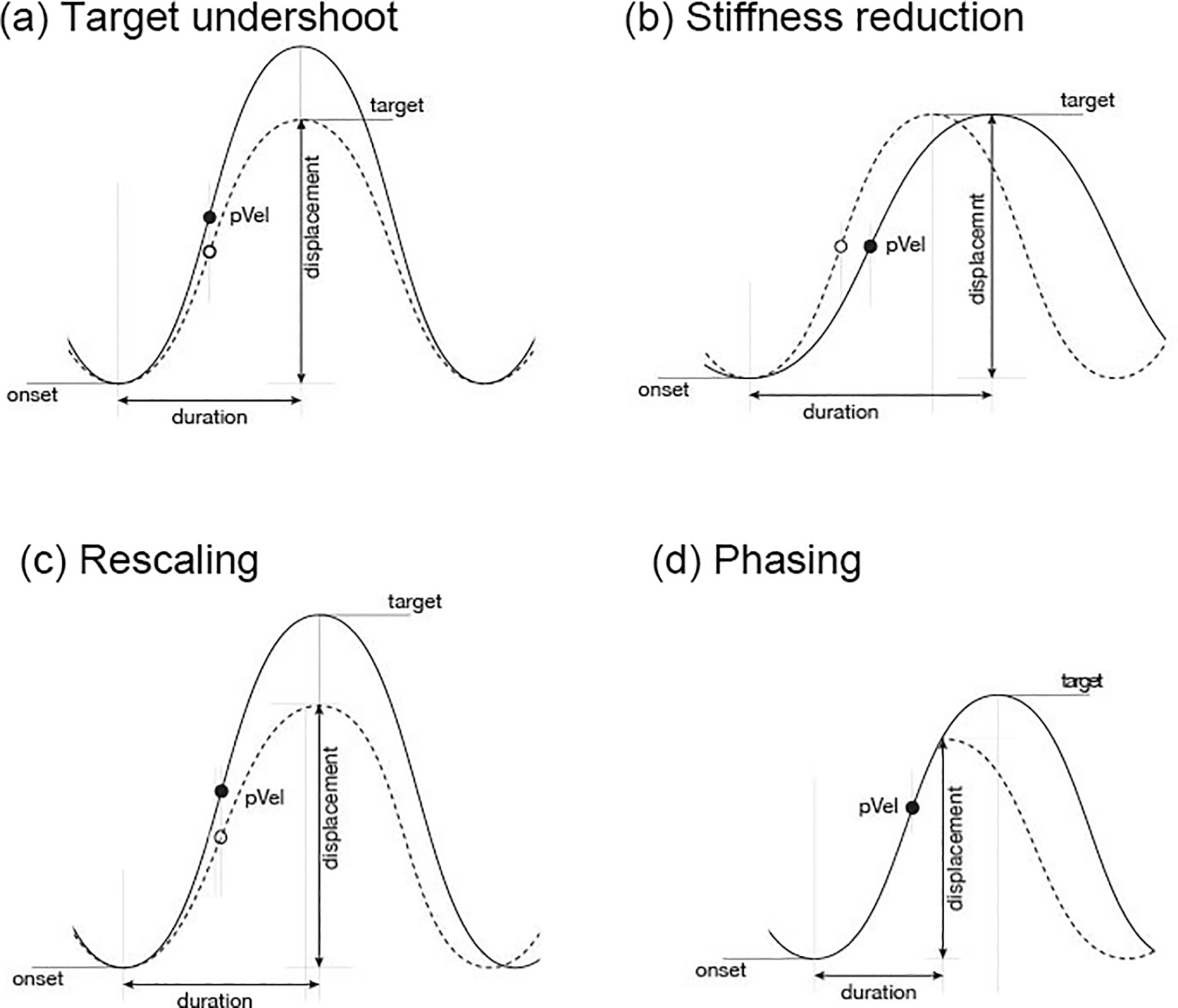
Schematised modification of articulatory control parameters.

Variations in an articulatory movement have direct consequences on abstract parameter settings underlying the observable kinematic pattern and therefore on the acoustic outcome [24–28]. These consequences involve the following possible parameter modifications: (a) target, (b) stiffness, (c) rescaling, and (d) phasing (presented in Table 2 and Fig 4). Note, that different parameter modifications can lead to the same effects in the acoustic domain, i.e., there are multiple solutions for achieving–and for not achieving–a desired motor goal [29].

**Table 2:** Modification of articulatory control parameters and related phonetic output.

Parameter	Parameter description and phonetic output
*(a) Target*	A change in the underlying target involves changes in the peak velocity in proportion to the target value (target undershoot), while the duration of the movement remains unchanged [30]. *Articulatory output*: A reduction in target in fast speech involves smaller and slower, but not shorter movements (target undershoot). *Acoustic output*: Reduction in quality, e.g. spirantization or centralisation.
*(b) Stiffness*	Stiffness is an abstract control parameter related to the relative speed of the movement. It is calculated as the ratio of peak velocity to the maximum displacement, a temporal-spatial measure [27,31–33] *Articulatory output*: Increasing a gesture’s underlying stiffness in fast speech leads to faster and shorter movements, i.e., the target is achieved in a shorter time. *Acoustic output*: Shorter durations
*(c) Rescaling*	Rescaling involves a proportional change in target and stiffness modifications. It affects the acceleration and deceleration phases [34] *Articulatory output*: Movements are shorter and smaller in fast speech, while the peak velocity remains the same. *Acoustic output*: Shorter duration and reduction in quality, e.g., spirantization or centralisation
*(d) Phasing*	Phasing affects the overlap between two gestures, e.g., the timing between a closure and a release. When the release gesture is timed earlier with respect to the closure, the overlap between the gestures increases and the closure will be truncated [27,35,36]. *Articulatory output*: In fast speech, the closing gesture becomes shorter (truncation, especially of the deceleration phase) and the target is reduced (undershoot), while the peak velocity remains the same. *Acoustic output*: Shorter duration and reduction in quality, e.g., spirantization or centralisation

### Statistical analysis

For all measures, all data were analyzed using R [37] and the package lme4 [38]. To analyze the continuous dependent variables, mixed linear regression models were fitted to scaled log-transformed (1) syllable duration data and (2) voicing-to-syllable ratio data, (5) log-transformed acceleration phase, (6) log-transformed deceleration phase, (7) log-transformed displacement, (8) log-transformed peak velocity, and (9) stiffness.

To analyze categorical data, mixed logit models with a binomial error function [39] were fitted to the binomial data of (3) voicing-during-closure and (4) closure frication-during-closure. The critical predictors were DBS (Control vs. DBS-OFF or DBS-OFF vs. DBS-ON) and POA (/pa, ta, ka/). Moreover, we included the control predictor syllable position within cycles (1–10, centered). Contrasts were deviation coded.

The random effects component included random intercepts for speakers. We refrained from using random slope structures due to convergence difficulties of the optimization process. The speaker-specific patterns with regard to the investigated parameters are summarized in the S1 Appendix for inspection.

In our model selection process, we tested whether including an interaction between DBS and POA significantly improved the model predictions. If there was a significant interaction, we concluded that there is a joint effect of DBS and POA. We validated the models by comparing the test model (with the/a critical predictor/interaction) to a reduced model (without the/a critical predictor/interaction) via likelihood-ratio tests. P-values are based on these comparisons. Since we tested multiple measurements for both the acoustic and articulatory parameters against the null hypothesis, we corrected for multiple testing using the Dunn–Šidák correction for both the acoustic and articulatory parameters separately. We measured four acoustic parameters lowering the analysis wide alpha level to 0.0127 and we measured five articulatory parameters lowering the analysis wide alpha level to 0.0102. In line with standards of reproducible research [40], the data tables and the scripts for the statistical analyses are made available and can be retrieved here: https://github.com/troettge/Muecke-et-al-Thalamic-Deep-Brain-Stimulation-changes-speech-dynamics.

## Results

### Characterization of patients’ tremor suppression and speech impairment

**Fig 5:**
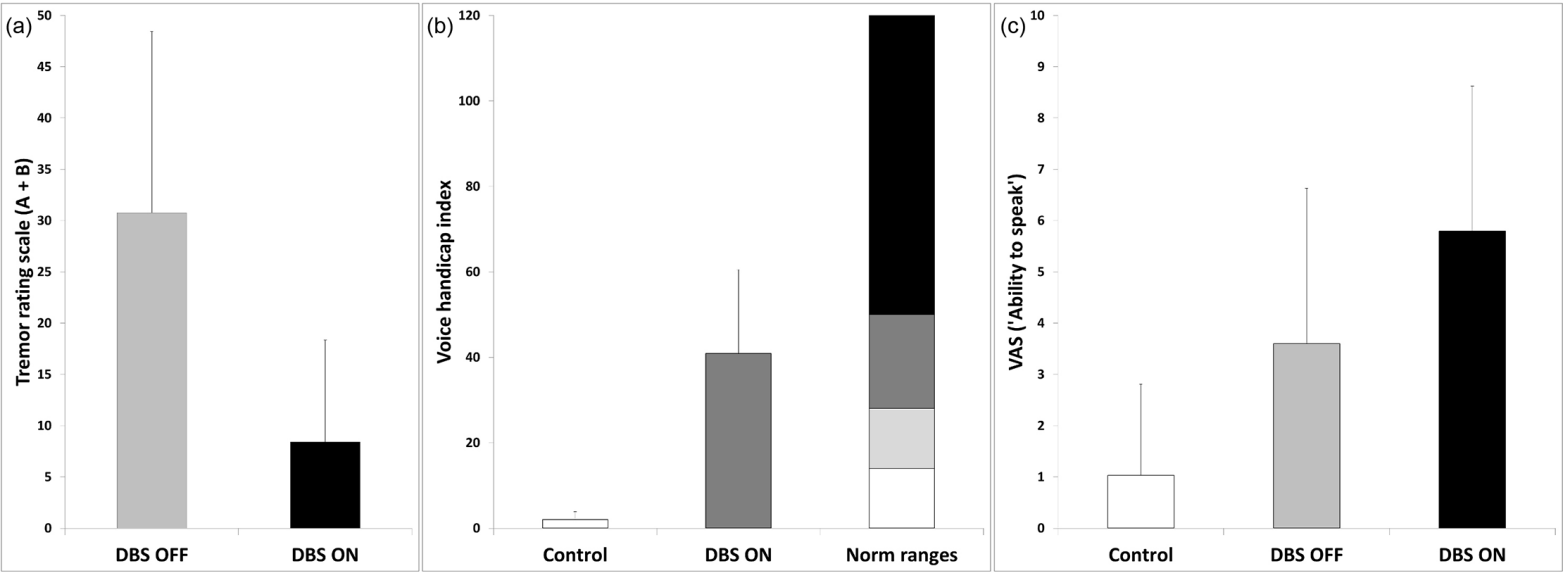
(a) TRS (A+B) scores for patients with inactivated (DBS-OFF) and activated stimulation (DBS-ON). (b) Mean VHI scores for control speakers and patients with DBS-ON according to VHI norm ranges. (c) VAS scores (“ability to speak”) for controls and patients with DBS-OFF and DBS-ON. Error bars represent standard deviation.

Patients’ TRS A + B scores improved while DBS was ON compared to the OFF-state (mean score of 30.75 (SD = 17.7) during DBS-OFF versus 8.42 (SD = 9.93) during DBS-ON), Fig 4A. For the VHI, there was a relevant speech impairment in the patient group (mean = 40.92, SD = 19.56, according to VHI norm ranges in line with moderate speech impairment) compared to the control group (mean = 2, SD = 1.85; indicating no handicap), Fig 5B. In line with this, the VAS scores for the control speakers were low (mean = 1.03, SD = 1.78), increasing for patients with inactivated DBS (DBS-OFF: mean = 3.6, SD = 3.03) with further worsening after DBS activation (DBS-ON: mean = 5.79, SD = 2.83), Fig 5C.

### Acoustic results

Table 3 presents the results for the acoustic measures analyzed, (1) syllable duration, (2) voicing-to-syllable ratio, (3) frication-during-closure, and (4) voicing-during-closure across subjects with means and standard deviations in parentheses, given separately for place of articulation (POA: /pa, ta, ka/) and DBS (control, OFF, ON).

**Table 3:** Acoustic measurements across subjects (mean and SD) for control group and ET patients with DBS-OFF and DBS-ON, sorted by place of articulation (/pa, ta, ka/).

Acoustic variable	POA	Control	DBS-OFF	DBS-ON
(1) Syllable duration (ms)	pa	151 (15)	193 (55)	221 (67)
	ta	163 (19)	217 (77)	242 (77)
	ka	173 (22)	229 (68)	273 (81)
(2) Voicing-to-syllable ratio (%)	pa	0.51 (0.11)	0.58 (0.17)	0.6 (0.14)
	ta	0.57 (0.12)	0.62 (0.15)	0.65 (0.15)
	ka	0.52 (0.1)	0.54 (0.14)	0.62 (0.15)
(3) Voicing-during-closure (%)	pa	0.44 (0.5)	0.66 (0.48)	0.72 (0.45)
	ta	0.48 (0.5)	0.58 (0.5)	0.84 (0.37)
	ka	0.26 (0.44)	0.38 (0.49)	0.53 (0.5)
(4) Frication-during-closure (%)	pa	0.05 (0.23)	0.19 (0.40)	0.33 (0.47)
	ta	0.65 (0.48)	0.65 (0.48)	0.86 (0.35)
	ka	0.26 (0.44)	0.57 (0.50)	0.75 (0.44)

**Fig 6:**
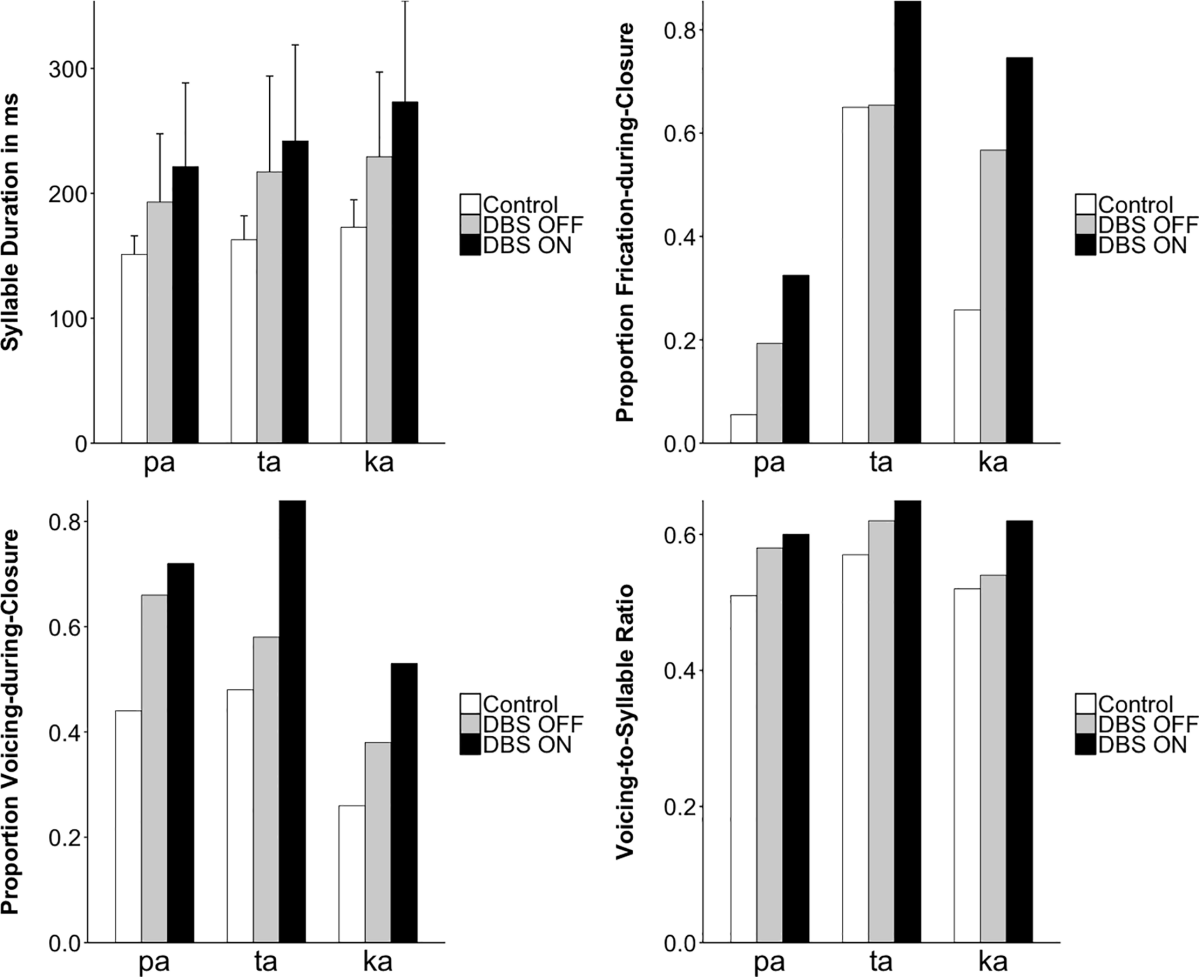
Acoustic measurements across subjects (mean and SD) for control speakers and ET patients with DBS-OFF and DBS-ON, shown separately for place of articulation (/pa, ta, ka/). The display of frication-during-closure and voicing-during-closure refers to the proportion of cases of which frication and/or voicing during the consonantal constriction phase in /p, t, k/ occurred. Means for the proportion and ratio measures are not displayed with error bars, since means are correlated with variability.

Fig 6 graphically displays the corresponding barplots (controls: white bars; DBS-OFF: grey bars; DBS-ON: black bars). Results are discussed below comparing controls vs. DBS-OFF and DBS-OFF vs. DBS-ON for the different places of articulation (/pa, ta, ka/) in the DDK task.

In all acoustic measures, standard deviations were also considerably higher when comparing controls with ET patients in DBS-OFF condition and when comparing ET patients in DBS-OFF with DBS-ON condition (Table 3).

Statistical analyses revealed the following main results:

(A) Overall speaking rate: Syllables were lengthened when comparing controls with ET patients in the DBS-OFF condition and when comparing ET patients during DBS-OFF with DBS-ON.
(B) Frication-during-closure: There was an increase in cases of frication for /pa/ and /ka/ when comparing controls with DBS-OFF and for all places of articulation when comparing DBS-OFF with DBS-ON.
(C) Voicing-during-closure: When comparing DBS-ON with DBS-OFF, there was an increase in voicing during the consonantal closure as well as an increase in voicing across the entire syllable cycle.

The results are reported in detail below, separately for the comparisons of the control group and ET patients with inactivated stimulation (DBS-OFF), and within-subject productions with inactivated and activated stimulation (DBS-OFF and DBS-ON).

### Comparing the control group to patients with inactivated stimulation (DBS-OFF)

(1) For **syllable duration**, there was a significant difference between control and DBS-OFF. More specifically, the analysis of syllable duration (CV = pa, ta, ka) revealed a significant interaction between DBS and POA (χ2(2) = 10.3; p = 0.0058). All three places of articulation, POAs, showed the same pattern numerically, i.e., longer syllables for the patients in the OFF condition (DBS-OFF: /pa/ = 193 ms, /ta/ = 217 ms, /ka/ = 229 ms) compared to the control group (Control: /pa/ = 151 ms, /ta/ = 163 ms, /ka/ = 173 ms). The differences between groups increased from /pa/ through /ta/ to /ka/. Patients with inactivated stimulation (DBS-OFF) showed an increase in syllable duration when compared with an age-matched group of healthy control speakers.
(2) For **voicing-to-syllable ratio,** there was neither a main effect of DBS (ß = 0.03, SE = 0.04, χ2(1) = 0.6; p = 0.43) nor a significant interaction between DBS and POA (χ2(2) = 1.6; p = 0.44).
(3) For **voicing-during-closure,** there was neither a significant interaction between DBS and POA (χ2(2) = 1.4; p = 0.49) nor a significant main effect of DBS (ß = 0.8, SE = 0.6, χ2(1) = 1.4; p = 0.23). However, numerically there was a trend for patients to exhibit larger proportions of instances with voicing-during-closure (0.54) than controls (0.39).
(4) For the parameter **frication-during-closure,** there was a significant interaction between DBS and POA (χ2(2) = 24.4; p < 0.0001). All three POAs showed the same pattern numerically, i.e., more frequent occurrences of frication during closure in DBS patients (DBS-OFF: /pa, ta, ka/ = 0.19, 0.65, 0.57) than for the control group (0.05, 0.65, 0.26). While for /pa/ and /ka/ the proportion of stops exhibiting frication during closure was larger in patients (/pa/ 0.19, /ka/ 0.57) than in controls (/pa/ 0.05, /ka/ 0.26), there was no such difference between groups for /ta/ (0.65 vs. 0.65).

### Comparing ET patients in two conditions, with activated (DBS-ON) and inactivated stimulation (DBS-OFF)

(1) For the **syllable duration,** there was a significant interaction between DBS and POA (χ2(1) = 15.8; p = 0.0004). All three POAs showed the same pattern numerically, i.e., longer syllable duration in patients with DBS-ON (/pa/ = 221 ms, /ta/ = 242 ms, /ka/ = 273 ms) than with DBS-OFF (/pa/ = 193 ms, /ta/ = 217 ms, /ka/ = 229 ms), with /ka/ showing the strongest effect followed by /pa/ and /ta/.
(2) For **voicing-to-syllable ratio,** there was a significant main effect of DBS (ß = 0.052, SE = 0.008, χ2(1) = 36.5; p < 0.0001) with greater proportions of voicing during the syllable cycles for DBS-ON (0.63) than DBS-OFF (0.58). There was no significant interaction of DBS and POA (χ2(2) = 5.2; p = 0.08).
(3) For **voicing-during-closure** there was no significant interaction of DBS and POA (χ2(2) = 7.9; p = 0.02, not significant after correction). There was a significant main effect of DBS (ß = 0.9, SE = 0.19, χ2(1) = 23.8; p < 0.0001) with greater proportions of voicing during closure for DBS-ON (0.69) than DBS-OFF (0.54).
(4) For **frication-during-closure,** there was a significant main effect of DBS (ß = 1.31, SE = 0.21, χ2(1) = 41.1; p < 0.0001) with greater proportions of frication during closure for DBS-ON (0.64) than DBS-OFF (0.47). There was no significant interaction between DBS and POA (χ2(2) = 3.5; p = 0.17).

### Articulatory results

Table 4 presents the results for the articulatory variables describing the consonantal gesture, (5) duration of the acceleration phase, (6) duration of the deceleration phase, (7) maximum displacement, (8) peak velocity, and (9) stiffness, across subjects for the three different groups, sorted by POA.

**Table 4:** Articulatory measurements (mean and SD) across subjects for control, DBS-OFF, and DBS-ON, sorted by POA.

Articulatory variable	POA	Control	DBS-OFF	DBS-ON
(5) Acceleration phase (ms)	pa	32 (5)	45 (23)	59 (42)
	ta	30 (5)	46 (24)	50 (28)
	ka	42 (8)	59 (30)	74 (37)
(6) Deceleration phase (ms)	pa	44 (7)	57 (17)	65 (24)
	ta	50 (9)	68 (26)	74 (33)
	ka	47 (8)	60 (18)	68 (24)
(7) Displacement (mm)	pa	4.9 (1.9)	6.9 (2.9)	5.8 (2.5)
	ta	3.9 (1.7)	5.6 (2.5)	4.5 (2.1)
	ka	4.1 (1.9)	5.2 (1.9)	4.8 (1.9)
(8) Peak velocity (mm/s)	pa	95.9 (35.9)	120.4 (43.5)	87.7 (31.7)
	ta	83.6 (33.4)	100.9 (36)	72.4 (29)
	ka	69.9 (31.2)	76.7 (29.4)	(24.1)
(9) Stiffness (pVel/displ)	pa	19.5 (1.7)	18.1 (4.3)	16 (4.8)
	ta	21.7 (2.4)	19.3 (4.9)	17.4 (5.4)
	ka	17.2 (1.8)	15 (3.1)	13.4 (3.6)

**Fig 7:**
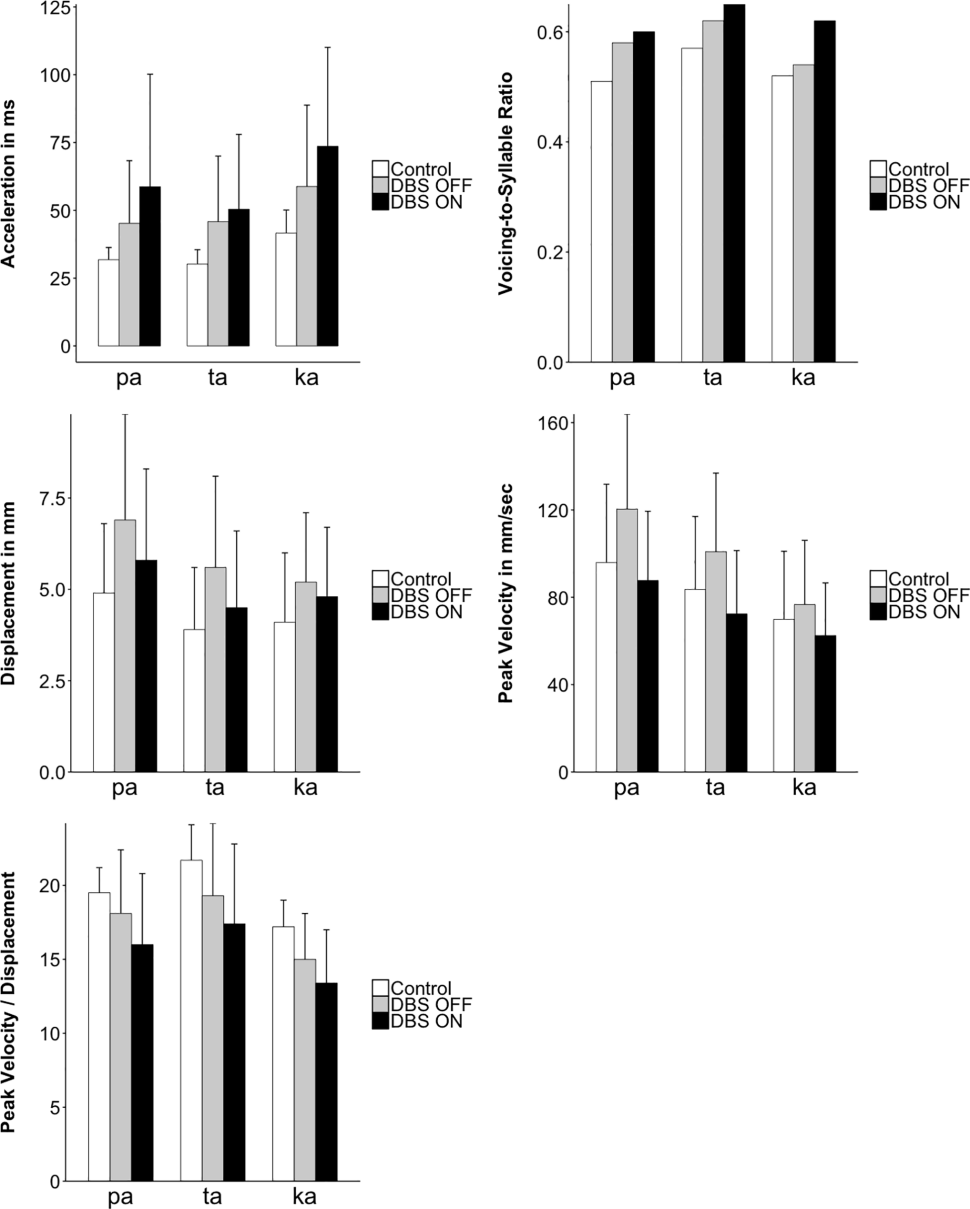
Articulatory measurements across subjects (mean and SD) for control group and ET patients with DBS-OFF and DBS-ON, shown separately for place of articulation (/pa, ta, ka/).

The aggregated results are further displayed in Fig 7 (control: white bars; DBS-OFF: grey bars; DBS-ON: black bars) for the different POAs. Results are discussed below by comparing controls vs. DBS-OFF and DBS-OFF vs. DBS-ON for the different places of articulation (/pa, ta, ka/) in the DDK task.

Analogously to the acoustic results, standard deviations were considerably higher when comparing controls with ET patients in DBS-OFF condition and when comparing ET patients during DBS-OFF versus DBS-ON (Table 4).

The statistical analysis showed the following main results for the consonantal gesture:

(A) Acceleration and deceleration phases increased when comparing controls with DBS-OFF and also when comparing DBS-OFF with DBS-ON.
(B) Stiffness decreased when comparing controls with DBS-ON and also when comparing DBS-OFF with DBS-ON.
(C) Peak velocity and displacement decreased when comparing DBS-OFF with DBS-ON.

As for the acoustic results, articulatory results are reported in detail below, separately for the comparisons of the control group and ET patients with inactivated stimulation (DBS-OFF), and within-subject productions with inactivated and activated stimulation (DBS-OFF and DBS-ON).

### Comparing the control group to patients with inactivated stimulation (DBS-OFF)

(5) For the parameter **acceleration phase,** there was a significant interaction between DBS and POA (χ2(2) = 9.7; p = 0.0079). All three POAs showed the same pattern numerically, i.e., longer acceleration phases for the patients in DBS-OFF (/pa/ = 45 ms, /ta/ = 46ms, /ka/ = 59 ms) than for the control group (/pa/ = 32 ms, /ta/ = 30 ms, /ka/ = 42 ms). However, the differences between groups increased from /pa/ through /ta/ to /ka/.
(6) Analysis of the **deceleration phase,** parameter revealed a significant main effect of DBS (ß = 0.76, SE = 0.24, χ2(1) = 8.3; p = 0.0039) with longer deceleration phases for the patients in the DBS-OFF condition (61 ms) than for the control group (47 ms). There was no significant interaction between DBS and POA (χ2(2) = 3.2; p = 0.19).
(7) For the parameter **displacement,** there was neither a significant main effect of DBS (ß = 0.68, SE = 0.27, χ2(1) = 5.5; p = 0.019, not significant after correction) nor a significant interaction between DBS and POA (χ2(2) = 6.5; p = 0.038).
(8) For **peak velocity,** there was neither a significant main effect of DBS (ß = 0.41, SE = 0.25, χ2(1) = 2.5; p = 0.11) nor a significant interaction between DBS and POA (χ2(2) = 2.2; p = 0.34) nor
(9) For the abstract control parameter **stiffness**, there was a significant interaction between DBS and POA (χ2(2) = 10.3; p = 0.0057). All three POAs showed the same pattern numerically, i.e., less stiffness for the DBS patients (/pa/ = 18 mm, /ta/ = 19 mm, /ka/ = 15 mm) than for the control group (/pa/ = 20 mm, /ta/ = 22 mm, /ka/ = 17 mm). The difference between groups increased from /ka/ through /pa/ to /ta/.

### Comparing ET patients in two conditions, with activated (DBS-ON) and inactivated stimulation (DBS-OFF)

(5) For the analysis of the parameter **acceleration phase** there was a significant main effect of DBS (ß = 0.37, SE = 0.05, χ2(1) = 52.2; p < 0.0001) with patients in the DBS-ON condition (60 ms) exhibiting longer acceleration phases than in the DBS-OFF condition (50 ms). There was no significant interaction between DBS and POA (χ2(2) = 6.7; p = 0.035).
(6) Analysis of the parameter **deceleration phase** revealed a significant main effect of DBS (ß = 0.31, SE = 0.05, χ2(1) = 37.6; p < 0.0001) with patients in the DBS-ON condition (69 ms) exhibiting longer deceleration phases than in the DBS-OFF condition (61 ms). There was no significant interaction between DBS and POA (χ2(2) = 0.5; p = 0.77).
(7) For **displacement,** there was a significant main effect of DBS (ß = 0.36, SE = 0.06, χ2(1) = 37.3; p < 0.0001) with patients in the DBS-ON condition (5.1 mm) exhibiting smaller displacement than in the DBS-OFF condition (6 mm). There was no significant interaction between DBS and POA (χ2(2) = 5.3; p = 0.07).
(8) Analysis of the **peak velocity** parameter revealed a significant main effect of DBS (ß = 0.65, SE = 0.06, χ2(1) = 104.1; p < 0.0001) with patients in the DBS-ON condition (75 mm/s) exhibiting lower peak velocity than in the DBS-OFF condition (101 mm/s). There was no significant interaction between DBS and POA (χ2(2) = 5; p = 0.08).
(9) For **stiffness,** there was a significant main effect of DBS (ß = 0.42, SE = 0.05, χ2(1) = 68.2; p < 0.0001) with patients in the DBS-ON condition (16) exhibiting lower stiffness than in the DBS-OFF condition (18). There was no significant interaction between DBS and POA (χ2(2) = 1.2; p = 0.55).

For all acoustic and articulatory measures, standard deviations were considerably higher for ET patients in the DBS-OFF condition compared to controls, and for patients in the DBS-ON condition compared to the DBS-OFF condition, pointing in the direction of a decrease in the control of articulatory forces that can be described as striking changes in the dynamics of the speech motor system.

**Fig 8:**
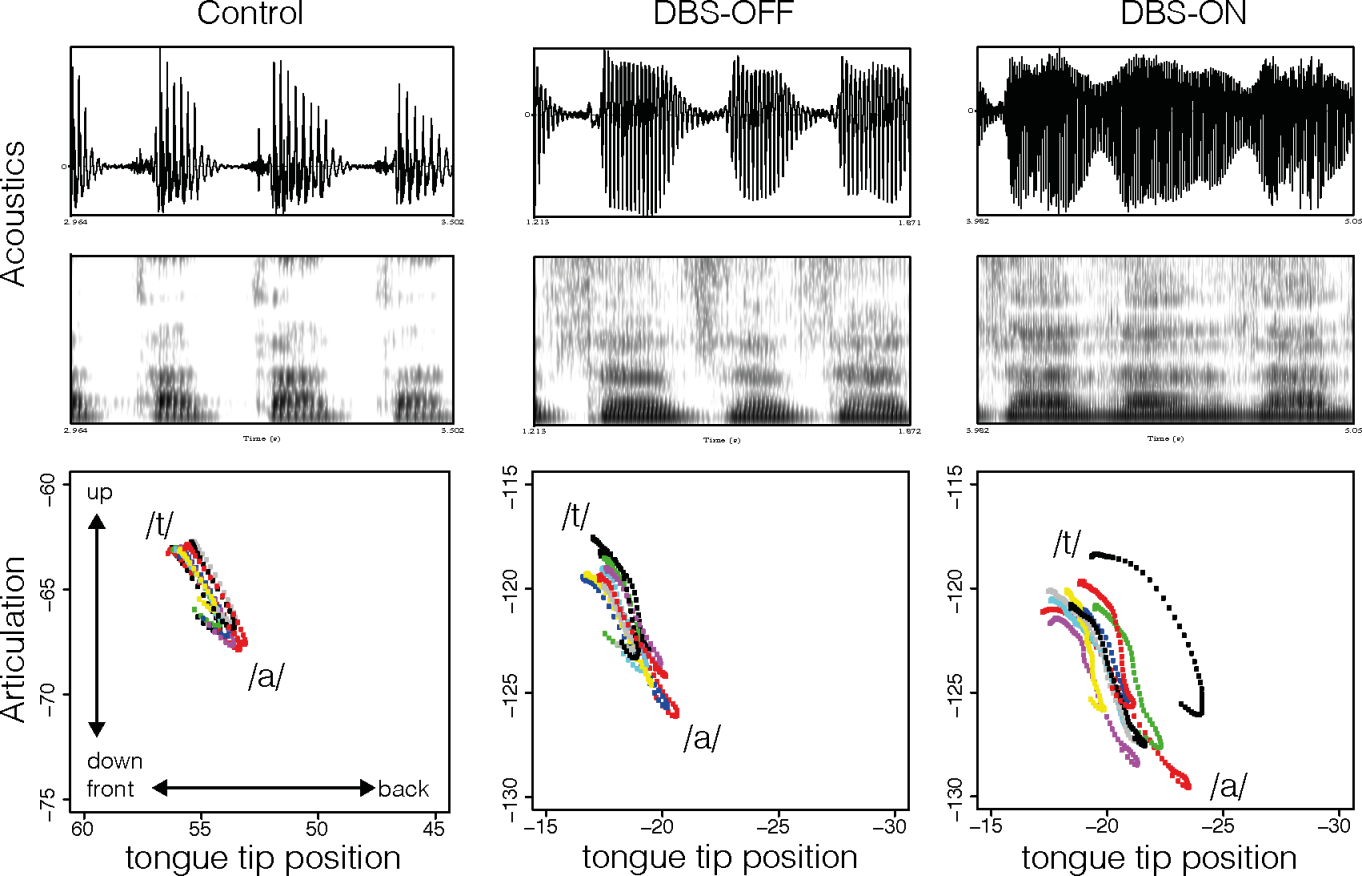
Acoustic waveform and spectrogram for /ta/ and corresponding tongue tip position (vertical and horizontal position with the same range across speakers) during the production of /t/, for one ET patient in the DBS-OFF (middle) and DBS-ON condition (right) and one age-matched control-speaker (left).

Fig 8 exemplifies the production of /t/ in the syllables /ta/ for one ET patient with inactivated stimulation (middle) and activated stimulation (right), and one age-matched healthy control speaker (left) in the acoustic and articulatory dimension. From top to bottom the figure displays the acoustic waveform with the respective spectrogram and the articulatory tongue tip position for the gestural activation interval of /t/, i.e., from start to target of the movement. The vertical axis displays the raising and lowering of the tongue and the horizontal axis displays the fronting and retracting of the tongue tip. During the production of /t/ the tongue tip is raised and fronted towards the alveolar ridge. The figure shows a progressive increase in voicing (periodic energy during the closure phase) and frication (aperiodic energy during the closure phase) on the acoustic surface accompanied by a higher degree of variability in tongue tip movement from the healthy control speaker through the DBS-OFF to the DBS-ON condition. When comparing the tongue tip positions for the patient with activated and inactivated stimulation, the maximum displacement values were lower, pointing to the fact that a higher number of leaking closures were produced under stimulation.

**Fig 9:**
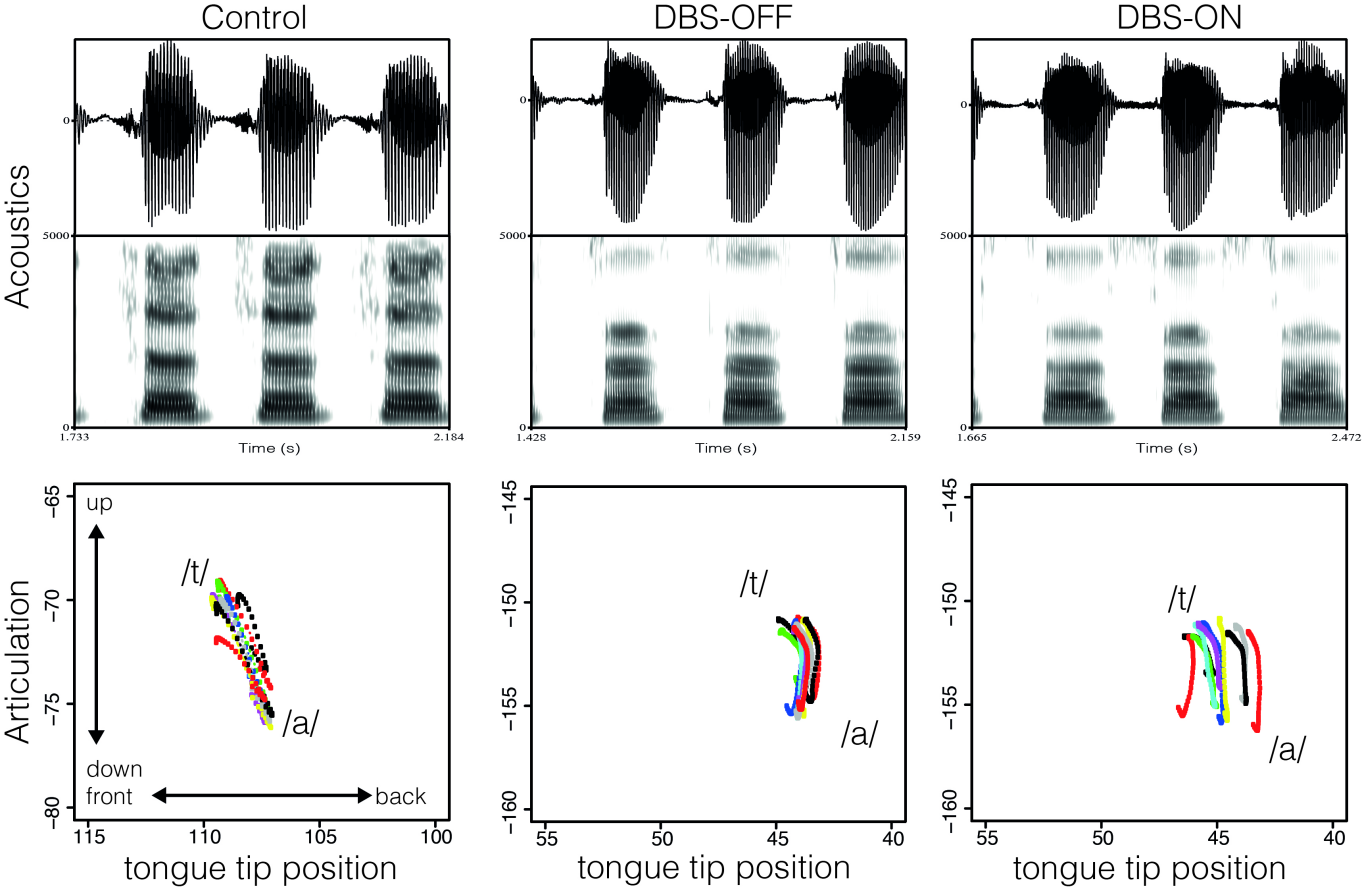
Acoustic waveform and spectrogram for /ta/ and corresponding tongue tip position (vertical and horizontal position with the same range across speakers) during the production of /t/, for one ET patient in the DBS-OFF (middle) and DBS-ON condition (right) and one age-matched control-speaker (left).

However, there was a high degree of speaker-specific variation and not all speakers were affected in the same way in DBS-OFF and DBS-ON condition. Fig 9 presents the tongue tip movement for another age-matched control speaker and corresponding patient in the DBS-OFF and DBS-ON conditions. Variability in the patient’s production increased under stimulation, but for this speaker this did not necessarily lead to target undershoot.

### Order effects of phenotypical assessments

EMA recordings were made after stimulation changes had been kept constant for a minimum of 20 minutes. As the sensors had to be kept on the articulators between the two recordings, longer intervals between the DBS-ON and DBS-OFF measurements were not possible due to the increasing risk of loosening of the sensors. However, one might argue that longer intervals between switching DBS on or off (e.g. 30 minutes) would have created more stable DBS effects because more time is needed to develop a stable DBS state. Due to this methodological decision, the timing between the different phenotypical assessments might have affected our measurements (as a reviewer rightfully pointed out).

To explore this possibility, we ran a series of additional model comparisons adding the relevant covariate of DBS order (ON-OFF vs OFF-ON). These additional analyses can be retrieved together with the data set here: https://github.com/troettge/Muecke-et-al-Thalamic-Deep-Brain-Stimulation-changes-speech-dynamics. Before reporting our analyses and their results, two caveats are in order. First, testing the impact of this covariate was not planned as part of our hypothesis evaluation. It is an exploratory (as opposed to hypothesis testing) post-hoc analysis that should be interpreted with caution and can only be taken as hypothesis generating (as opposed to hypothesis testing). Second, and related to the first caveat, because this co-variate was originally not intended to be tested, our experimental design including our chosen sample size did not take this covariate into account. Albeit counter-balanced the order of DBS, we end up with a small number of participant for each order group. The resulting low power increases both Type I and Type II errors.

As a starting point, we took the specified models of the main analysis (see section on acoustic and articulatory results) without the interaction terms between DBS and POA to simplify our interpretation. For the comparisons between control group vs. patients in DBS-OFF, we tested whether a model with DBS order (i.e. control vs. OFF (order OFF-ON) vs. OFF (order ON-OFF) does improve the model fit significantly compared to a model with DBS only (i.e. control vs. OFF). For the comparison between DBS-OFF and DBS-ON, we tested whether there is either an interaction of DBS order and DBS or (if the interaction term was significant) a main effect of DBS order. Table 5 presents the descriptive means for the acoustic and articulatory parameters related to the order of DBS (ON-OFF vs. OFF-ON) including effects between groups.

**Table 5:** Descriptive means and effects between groups.

	Control	DBS-OFF		DBS-ON		Model comparison	
		OFF-ON	ON-OFF	OFF-ON	ON-OFF	Order effect in C vs Off	Order effect in ON vs Off
(1) Syllable duration (ms)	162.58	180.34	239.62	192.57	283.79	only ON-OFF differs from C	interaction with DBS
(2) Voicing-to-syllable ratio (%)	0.53	0.64	0.53	0.64	0.62	ns	interaction with DBS
(3) Voicing-during-closure (%)	0.39	0.65	0.44	0.74	0.66	ns	ns
(4) Frication-during-closure (%)	0.33	0.58	0.38	0.67	0.62	ns	interaction with DBS
(5) Acc. phase (ms)	34.76	44.52	53.24	46.88	70.23	ns	interaction with DBS
(6) Dec. phase (ms)	47.06	58.11	63.76	65.01	71.92	ns	ns
(7) Displacement (mm)	4.32	6.16	5.79	5.35	4.87	ns	ns
(8) Peak velocity (mm/s)	82.74	110.61	93.43	88.48	64.85	ns	interaction with DBS

The analysis indeed reveals an order effect of phenotypical assessment and corresponding interactions in our dataset.

For the comparison between Control and DBS-OFF, only the acoustic measure of syllable duration indicates a main effect of DBS order, with only those patients that arrived in a DBS-ON state showing a significant difference (in their DBS-OFF state) compared to the control (χ2(1) = 6.2; p = 0.013).

The differences between ET patients with activated and inactivated stimulation are considerably larger when they went from DBS-ON to DBS-OFF, compared to when they went from DBS-OFF to DBS-ON, as indicated by a significant interaction term between DBS order and DBS stimulation. This interaction term turned out to be significant for syllable duration (χ2(1) = 24; p<0.0001), frication during closure (χ2(1) = 8.3; p = 0.004), voicing-to-syllable-ratio (χ2(1) = 31.5; p<0.0001), acceleration (χ2(1) = 17.1; p<0.0001), as well as peak velocity (χ2(1) = 6.7; p = 0.001).

We conclude that for some dependent measures, there is a strong effect of the stimulation order, which could inform the experimental design of future studies. However, as far as the available evidence suggests, the obtained differences between DBS-ON and DBS-OFF remain intact regardless of the stimulation order.

## Discussion

### Limitations of the study

Basically, three limitations have to be considered in this study before we providing an interpretation of the results. First and most relevant, we are aware that speech in ET patients with inactivated DBS-electrodes (DBS-OFF) is not identical to speech in DBS-naive ET patients. Of course, we cannot exclude that the pure presence of the inactivated electrodes causes the observed changes in the speech motor system. Thus, our study design does not allow to differentiate whether the observed changes in speech motor control in ET patients with DBS-OFF are due to a microlesional effect of the electrode or whether they are a sign of subclinical dysarthria induced by the Essential Tremor itself (or a combination of both) when being compared to healthy controls. However, since Kronenbuerger et al. [7] provided evidence that speech in DBS-naïve ET patients is already deteriorated compared to healthy controls, we assume that the observed effect in DBS-OFF condition can be at least partially attributed to subclinical dysarthria rather than being solely explained by the mere presence of the inactivated electrodes. For further clarification, a larger-scale EMA-study is needed, comparing DBS-naïve ET patients with and without cerebellar signs to an age- and sex-matched control group.

Second, we found order effects due to methodological decisions. EMA recordings were made after stimulation changes had been kept constant for a minimum of 20 minutes. However, slightly longer intervals are recommended for the patients to adapt to the different phenotypical assessments [41] and this should be taken into account for the test design in future studies. Indeed, we found that differences between DBS-ON and DBS-OFF state were stronger for patients that arrived in DBS-ON. However, effects of different phenotypical assessment were also present when patients arrived in DBS-OFF state.

Finally, it is worth mentioning that fast syllable repetition tasks are not directly comparable to natural speech. When pushing the speech motor system to its limits one might discover qualitative changes of the speech motor system that are related to the requirements of the novel motor task rather than natural sentence production [42]. However, the DDK task has been used before in a variety of acoustic studies and thus allows for comparability of our data with previous results.

### Acoustic data

In a first step, we replicated the *acoustic results* from [8] thereby confirming that speech parameters deteriorate in ET patients, irrespective of stimulation being activated or inactivated. When comparing the control group with the ET patients in the inactivated (OFF) condition, we found prolonged *syllable durations* as a common feature for dysarthria [43–45]. This is in line with [7], who found that patients with advanced disease, i.e., with additional intention tremor, show slower articulation rates compared to those with postural tremor and healthy controls. However, [7] did not detect speech impairment under thalamic DBS. In contrast, our data suggest that articulation rate considerably slowed down under stimulation, i.e., there was an additional effect of VIM-DBS on the articulation rate when comparing ET patients with activated (DBS-ON) and inactivated stimulation (DBS-OFF). Within a gestural approach, speech deterioration in ET patients the slowing down of overall articulation rate is seen as a dynamic process that results from the increasing duration of consonantal and vocalic gestures and/or a decrease of overlap between them. Note moreover that previous studies have found a tendency for patients in DBS-ON to show increased syllable durations compared to DBS-OFF, but never vice versa [8].

Furthermore, we found an increase of *frication* during the constrictions of the consonants for /pa/ and /ka/ syllables when comparing controls with patients in DBS-OFF. In line with [8] for ET patients and [9] for multiple sclerosis (MS) patients, this effect worsened considerably under stimulation for all places of articulation within the patients’ group (DBS-OFF vs. DBS-ON). Frication is a type of spirantization, caused by incomplete closure in the vocal tract, and is considered to be a feature of dysarthric speech [22,43–47]. It results from the loss of control of articulatory force, leading to target under- or even overshoot (hypo- and hyperspeech). For example, the production of the stop consonant /t/ in <tea> requires a full closure of the tongue tip at the alveolar ridge in order to block the oral airflow leading to a silent gap on the acoustic surface. In case of an undershoot of the desired motor goal, i.e., in the case in which the articulatory closure is not fully achieved, air leaks out of the mouth during the closure phase. On the acoustic surface, frication will be generated (spirantization), shifting the phonetic cues to the stop consonant /t/ in <tea> towards a fricative /s/ as in <sea>. The phonetic specificity of the syllable onset is thus strongly reduced and speech intelligibility decreased [44].

The glottal system was also affected by the stimulation. When comparing the conditions DBS-ON and DBS-OFF, there was an increase of *voicing during the consonantal closure* as well as an *increase of voicing across the entire syllable cycle* shifting the voiceless stops in the direction of voiced stops. Insufficient glottal abduction is interpreted as a sign of dysarthric speech [8,9,44,47–49].

### Articulatory data

For the articulatory analysis, we investigated the duration of the gestural activation interval for the consonantal closure. Both the *acceleration* and *deceleration phases* were longer when comparing controls with patients with inactivated stimulation (DBS-OFF). The duration of the gestural activation interval for the consonant was considerably longer for patients in DBS-OFF compared to controls. This effect was even greater under activated stimulation.

In addition, the abstract control parameter *stiffness* decreased when comparing controls with patients in the DBS-OFF condition. Stiffness also decreased when comparing patients in the DBS-OFF and DBS-ON conditions. Stiffness was calculated by obtaining the ratio of displacement to maximum velocity, and pure changes in displacement were related to changes in relative speed. A decrease in stiffness should correlate with a decrease in relative speed, where there are slower and longer movements while the distance the articulator travels remains the same. However, this expectation was not confirmed by our data: since peak velocity and displacement did not change in a proportional way, our data rather suggest that we deal with a combination of stiffness and changes in overlap between gestures (gestural phasing), both contributing to syllable lengthening as well as the spirantization of stop consonants.

Furthermore, when looking at the data descriptively, the peak velocity values showed an increase (instead of a decrease) in the DBS-OFF group by an amount of 16mm/sec, compared to controls. In addition, the displacement values also showed a tendency to increase in the DBS-OFF group. There was a tendency for the patients’ group (DBS-OFF; power compared to controls) to expend a greater degree of biomechanical power. The ET patients with DBS-OFF were unable to minimize the articulatory effort in the fast motor task [50]. Even though there was a high degree of biomechanical power, the motor goals were not achieved (otherwise no frication would show up on the acoustic surface). This result is interesting, since the speech motor system usually tends to *minimize* the amount of articulatory effort for vocal tract movements during speech. Following the Hyperarticulation and Hypoarticulation (H&H) model developed by [51], speakers constantly vary along a continuum of over- and under-articulated speech (hyper- and hypo-articulated speech) in order to adapt to the complex demands of the communication process [52,53]. This leads to an increase in overlap between articulatory gestures and therefore to a higher degree of coarticulation, which is related to *hypo-speech*. In contrast, *hyper-speech* leads to a decrease in coarticulatory overlap and therefore to a more distinct articulation, which enhances distances in the perceptual space. Hyper-speech adds more biomechanical power and performance accuracy to a syllable or word to increase or decrease perceptual distances between competing words or syllables and therefore increases the associated resource costs [30,34,36,54–58]. Therefore, a fast syllable repetition task should lead into a minimization of biomechanical power, but the opposite was found in our patients’ data, suggesting that patients’ have difficulty controlling articulatory force [30,52]. One explanation for this observation might be the existence of cerebellar dysfunction observed in ET patients. Multiple studies reported cerebellar signs in ET patients, especially in the advanced stages of the disease. Symptoms include gait and balance disorders [59–61] as well as dysmetric eye movements [62,63].

When comparing *peak velocity* and *displacement* values for patients in inactivated and activated stimulation (DBS-OFF vs. DBS-ON), we observed a clear effect of stimulation. Both parameters decreased under stimulation, revealing an overall slowing down of the system, thus, a respective target undershoot. The movements were too slow to reach the desired articulatory goals in the given time frame leading to truncation. Under stimulation, patients showed an additional slowing-down of the system leading to a very poor performance on the syllable task. In terms of a mass-spring model, this can best be described as a combination of stiffness reduction and phasing variation leading to truncation of the consonantal movement (cf. Table 2 parameter modification).

The slowing-down increased the articulatory coordination problems. Note that the coordination of the speech motor system is already constrained in ET patients with inactivated stimulation (DBS-OFF). When the stimulation (DBS-ON) was activated, timing deficits of the lingual and labial systems worsened leading to an increase in lengthened syllables and spirantization on the acoustic surface, both interpreted as signs of dysarthria [22,43–46]. In addition, variability increased in all temporal and spatial articulatory parameters. These changes were not due to modification of a single parameter. We therefore assume that variation in stiffness in combination with changes in overlap between gestures (gestural phasing) contribute to syllable lengthening as well as the spirantization of stop consonants.

Basically, two pathophysiological mechanisms may explain stimulation induced dysarthria in ET patients treated with VIM-DBS. First, current spread to the motor fibers of the internal capsule may cause upper motor neuron or ‘spastic’ dysarthria in these patients [4,64]. This idea is supported by the fact that severity of dysarthria correlates with more laterally placed electrodes, which are closer to the internal capsule [65]. Another possible mode of action could be that stimulation induced dysarthria results from affection of the dentato-thalamic tract, which would result in cerebellar or atactic dysarthria. Several studies have shown that supra-therapeutic stimulation in ET patients can induce atactic symptoms such as gait ataxia or ataxia of the upper limbs [66,67].

However, overall slowing down could also be a compensatory mechanism, i.e., patients talking slower due to a DBS-induced deficit. As slowing down of the speech kinematics is a feature of both upper motor neuron dysarthria and cerebellar dysarthria [68], the kinematic data cannot offer further insights into the pathophysiology of stimulation induced dysarthria. Moreover, stimulation induced dysarthria may also derive from a combination of both mechanisms within the same patient. Also, the study population investigated here might be heterogeneous, i.e., some of our patients may have suffered from spastic dysarthria whilst others may have suffered from the atactic type. The aim of this study was to thoroughly analyze speech changes observed in VIM-DBS in ET-patients and not to differentiate between these possible origins. Future studies are now needed to especially investigate the relation between lead locations, stimulation volumes, individual surrounding anatomy, and changes in speech. Incorporating new imaging modalities like MRI tractography and fiber tracking [69] might provide new insights by being able to directly visualize neuroanatomical structures whose affection might lead to speech deterioration like the internal capsule or the dentate-thalamic tract.

## Conclusion

Articulatory and acoustic data suggest that we are dealing with two problems of the speech motor system when we investigate ET patients with activated and inactivated stimulation compared to healthy controls, namely a coordination deficit (DBS-OFF compared to controls) that worsened under thalamic deep brain stimulation. This increase in articulatory coordination problems likely triggered the additional overall slowing-down of the system that was found (DBS-ON compared to DBS-OFF). Articulatory imprecision and slowness leads to a poor performance in syllable production on the acoustic surface, reflecting an aggravation either of pre-existing cerebellar deficits and/or the affection of the upper motor fibers of the internal capsule under thalamic deep brain stimulation.

## Ethics

This study was approved by the Local Ethics Committee of the University of Cologne (14–301). Each participant gave written informed consent before study participation. Research was conducted in accordance with the Declaration of Helsinki. The individual in this manuscript has given written informed consent (as outlined in PLOS consent form) to publish these case details.

## Supporting information

S1 Appendix Stereotactic coordinates. Stereotactic coordinates from 24 electrodes with reference to the midcommissural point (MCP). Stimulation parameters: amplitude (V; mA), pulse duration (μsec) and stimulation frequency (Hz).
(PDF)

## References

[1] Louis ED, Ferreira JJ. How common is the most common adult movement disorder? Update on the worldwide prevalence of essential tremor. Movement Disorders. 2010 4 15;25(5):534–41. doi: 10.1002/mds.22838 20175185 10.1002/mds.22838

[2] Deuschl G, Elble R. Essential tremor—Neurodegenerative or nondegenerative disease towards a working definition of ET. Movement Disorders. Wiley-Blackwell; 2009 10;24(14):2033–41. 10.1002/mds.22755 19750493

[3] Schuurman PR, Bosch DA, Bossuyt PMM, Bonsel GJ, van Someren EJW, de Bie RMA, et al A Comparison of Continuous Thalamic Stimulation and Thalamotomy for Suppression of Severe Tremor. New England Journal of Medicine. New England Journal of Medicine (NEJM/MMS); 2000 2;342(7):461–8. doi: 10.1056/NEJM200002173420703 10675426 10.1056/NEJM200002173420703

[4] Krack P, Dostrovsky J, Ilinsky I, Kultas-Ilinsky K, Lenz F, Lozano A, et al Surgery of the motor thalamus: Problems with the present nomenclatures. Movement Disorders. Wiley-Blackwell; 2002 3;17(S3):S2–S8. 10.1002/mds.10136 11948749

[5] Flora Della E, Perera CL, Cameron AL, Maddern GJ. Deep brain stimulation for essential tremor: A systematic review. Movement Disorders. Wiley-Blackwell; 2010 7;25(11):1550–9. doi: 10.1002/mds.23195 20623768 10.1002/mds.23195

[6] Pahwa R, Lyons KE, Wilkinson SB, Simpson RK, Ondo WG, Tarsy D, et al Long-term evaluation of deep brain stimulation of the thalamus. Journal of Neurosurgery. Journal of Neurosurgery Publishing Group (JNSPG); 2006 4;104(4):506–12. doi: 10.3171/jns.2006.104.4.506 16619653 10.3171/jns.2006.104.4.506

[7] Kronenbuerger M, Konczak J, Ziegler W, Buderath P, Frank B, Coenen VA, et al Balance and Motor Speech Impairment in Essential Tremor. Cerebellum. Springer Nature; 2009 5;8(3):389–98. doi: 10.1007/s12311-009-0111-y 19452239 10.1007/s12311-009-0111-y

[8] Mücke D, Becker J, Barbe MT, Meister I, Liebhart L, Roettger TB, et al The Effect of Deep Brain Stimulation on the Speech Motor System. Journal of Speech, Language, and Hearing Research. American Speech-Language-Hearing Association; 2014;57(4):1206–18. doi: 10.1044/2014_JSLHR-S-13-0155 24686442 10.1044/2014_JSLHR-S-13-0155

[9] Pützer M, Barry WJ, Moringlane JR. Effect of Deep Brain Stimulation on Different Speech Subsystems in Patients with Multiple Sclerosis. Journal of Voice. Elsevier BV; 2007 11;21(6):741–53. doi: 10.1016/j.jvoice.2006.05.007 16872803 10.1016/j.jvoice.2006.05.007

[10] Hartinger M, Tripoliti E, Hardcastle WJ, Limousin P. Effects of medication and subthalamic nucleus deep brain stimulation on tongue movements in speakers with Parkinson's disease using electropalatography: A pilot study. 2010;25(3):210–30. doi: 10.3109/02699206.2010.521877 21158488 10.3109/02699206.2010.521877

[11] Chenausky K, MacAuslan J, Goldhor R. Acoustic Analysis of PD Speech. Parkinson's Disease. 2011;2011(4):1–13. 10.4061/2011/435232 PMC3185254 21977333

[12] Dromey C, Bjarnason S. A Preliminary Report on Disordered Speech with Deep Brain Stimulation in Individuals with Parkinson's Disease. Parkinson's Disease. Hindawi; 2011;2011(4):1–11. 10.4061/2011/796205 PMC3195307 22046577

[13] Karlsson F, Unger E, Wahlgren S, van Dorn J. Treatment effects in voice onset time of plosives associated with deep brain stimulation of the subthalamic nucleus and the caudal zona incerta. Journal of Medical Speech-Language Pathology 2012 12;20(4):65–69.

[14] Karlsson F, Olofsson K, Blomstedt P, Linder J, Nordh E, van Doorn J. Articulatory Closure Proficiency in Patients With Parkinson's Disease Following Deep Brain Stimulation of the Subthalamic Nucleus and Caudal Zona Incerta. Journal of Speech, Language, and Hearing Research. 2014 8 1;57(4):1178–13. doi: 10.1044/2014_JSLHR-S-13-0010 24686561 10.1044/2014_JSLHR-S-13-0010

[15] Fahn S, Tolosa E, Marín C. Clinical rating scale for tremor In: Jankovic J, Tolosa E, eds. Parkinson's disease and Movement Disorders. Baltimore: Williams & Wilkins; 1993 (2nd ed): 271–280. doi: 10.1002/mds.870080304

[16] Jacobson BH, Johnson A, Grywalski C, Silbergleit A, Jacobson G, Benninger MS, Newman CW. The voice handicap index (VHI): development and validation. American Journal of Speech-Language Pathology. 1997 8 1;6(3):66–70.

[17] Frost E, Tripoliti E, Hariz MI, Pring T, Limousin P. Self-perception of speech changes in patients with Parkinsons disease following deep brain stimulation of the subthalamic nucleus. International Journal of Speech-Language Pathology. Informa UK Limited; 2010 7;12(5):399–404. doi: 10.3109/17549507.2010.497560 20602580 10.3109/17549507.2010.497560

[18] Barbe MT, Liebhart L, Runge M, Pauls KA, Wojtecki L, Schnitzler A, Allert N, Fink GR, Sturm V, Maarouf M, Timmermann L. Deep brain stimulation in the nucleus ventralis intermedius in patients with essential tremor: habituation of tremor suppression. Journal of neurology. 2011 3 1;258(3):434–9. doi: 10.1007/s00415-010-5773-3 20927533 10.1007/s00415-010-5773-3

[19] Mai JK, Majtanik M, Paxinos G. Atlas of the human brain Academic Press; 2015 12 2.

[20] Fougeron C, Keating PA. Articulatory strengthening at edges of prosodic domains. The Journal of the Acoustical Society of America. Acoustical Society of America (ASA); 1997 6;101(6):3728–40. 9193060 10.1121/1.418332

[21] Boersma, P, Weenink, E. Praat: Doing phonetics by computer (Version 5.1.30) [Computer program]; 2010; Retrieved from http://www.praat.org/

[22] Weismer G. Acoustic Descriptions of Dysarthric Speech: Perceptual Correlates and Physiological Inferences. Seminars in Speech and Language. Thieme Publishing Group; 1984 11;5(04):293–314.

[23] Cassidy S, Harrington J. Multi-level annotation in the Emu speech database management system. Speech Communication. 2001 1 31;33(1):61–77.

[24] Saltzman, E. Task dynamic coordination of the speech articulators: A preliminary model. Status Report on Speech Research SR-84, Haskins Laboratories; 1975; 1–18.

[25] Browman CP, Goldstein L. Articulatory gestures as phonological units. Phonology. Cambridge University Press (CUP); 1989 8;6(02):201–51.

[26] Browman CP, Goldstein L. Articulatory Phonology: An Overview. Phonetica. 1992;49:155–80. 1488456 10.1159/000261913

[27] Saltzman E, Kelso JAS. Skilled actions: A task dynamic approach. Psychological Review. 1987;94:84–106. 3823306

[28] Beckman M, Edwards J, Fletcher J. Prosodic structure and tempo in a sonority model of articulatory dynamics In: Docherty GJ, Ladd DR, editors. Gesture, Segment, Prosody. Cambridge University Press; pp. 68–89.

[29] Patri J-F, Diard J, Perrier P. Optimal speech motor control and token-to-token variability: a Bayesian modeling approach. Biological Cybernetics. Springer Nature; 2015 10;109(6):611–26. doi: 10.1007/s00422-015-0664-4 26497359 10.1007/s00422-015-0664-4

[30] de Jong KJ. The supraglottal articulation of prominence in English: Linguistic stress as localized hyperarticulation. The Journal of the Acoustical Society of America. Acoustical Society of America (ASA); 1995 1;97(1):491–504. 7860828 10.1121/1.412275

[31] Munhall KG, Ostry DJ, Parush A. Characteristics of velocity profiles of speech movements. Journal of Experimental Psychology: Human Perception and Performance. 1985 8;11(4):457 3161986 10.1037//0096-1523.11.4.457

[32] Hawkins S. An introduction to task dynamics In: Docherty GJ, Ladd DR, editors. Gesture, Segment, Prosody. Cambridge University Press; 1992 pp. 9–25.

[33] Roon, KD, Gafos, AI, Hoole, P, & Zeroual, C. Influence of articulator and manner on stiffness. Proceedings 16th ICPhS 2007. 2007; 409–412.

[34] Harrington J, Fletcher J, Beckman M. Manner and place conflicts in the articulation of accent in Australian English In: Papers in Laboratory Phonology V Acquisition and the Lexicon. Cambridge University Press, London; 2000 pp. 40–51.

[35] Cho T. Manifestation of prosodic structure in articulatory variation: Evidence from lip kinematics in English. Laboratory phonology. 2006;8:519–48.

[36] Iskarous K, Kavitskaya D. The interaction between contrast, prosody, and coarticulation in structuring phonetic variability. Journal of phonetics. 2010 10 31;38(4):625–39. doi: 10.1016/j.wocn.2010.09.004 21218130 10.1016/j.wocn.2010.09.004 PMC3016051

[37] R Core Team. R: A language and environment for statistical computing R Foundation for Statistical Computing, Vienna, Austria; 2015; URL http://www.R-project.org/.

[38] Bates D, Maechler M, Bolker B, Walker S. lme4: Linear mixed-effects models using Eigen and S4_. R package version 1.1–7; 2014; URL http://CRAN.R-project.org/package=lme4.

[39] Jaeger TF. Categorical data analysis: Away from ANOVAs (transformation or not) and towards logit mixed models. Journal of memory and language. 2008 11 30;59(4):434–46. doi: 10.1016/j.jml.2007.11.007 19884961 10.1016/j.jml.2007.11.007 PMC2613284

[40] Peng RD. Reproducible Research in Computational Science. Science. American Association for the Advancement of Science (AAAS); 2011 12;334(6060):1226–7. doi: 10.1126/science.1213847 22144613 10.1126/science.1213847 PMC3383002

[41] Temperli P, Ghika J, Villemure JG, Burkhard PR, Bogousslavsky J, Vingerhoets FJG. How do parkinsonian signs return after discontinuation of subthalamic DBS? Neurology. Ovid Technologies (Wolters Kluwer Health); 2003 1;60(1):78–81. 12525722 10.1212/wnl.60.1.78

[42] Staiger A, Schölderle T, Brendel B, Bötzel K, Ziegler W. Oral Motor Abilities Are Task Dependent: A Factor Analytic Approach to Performance Rate. Journal of Motor Behavior. Informa UK Limited; 2016 12;49(5):482–93. doi: 10.1080/00222895.2016.1241747 27935471 10.1080/00222895.2016.1241747

[43] Ackermann H, Hertrich I, Hehr T. Oral Diadochokinesis in Neurological Dysarthrias. Folia Phoniatr Logop. S. Karger AG; 1995;47(1):15–23. 7728177 10.1159/000266338

[44] Kent RD, Weismer G, Kent JF, Vorperian HK, Duffy JR. Acoustic studies of dysarthric speech. Journal of Communication Disorders. Elsevier BV; 1999 5;32(3):141–86. 10382143 10.1016/s0021-9924(99)00004-0

[45] Ziegler W. Task-Related Factors in Oral Motor Control: Speech and Oral Diadochokinesis in Dysarthria and Apraxia of Speech. Brain and Language. Elsevier BV; 2002 3;80(3):556–75. doi: 10.1006/brln.2001.2614 11896657 10.1006/brln.2001.2614

[46] Logemann JA, Fisher HB. Vocal Tract Control in Parkinsons Disease. Journal of Speech and Hearing Disorders. American Speech Language Hearing Association; 1981 11;46(4):348 7300262 10.1044/jshd.4604.348

[47] Ziegler W, Cramon von D. Disturbed coarticulation in apraxia of speech: Acoustic evidence. Brain and Language. Elsevier BV; 1986 9;29(1):34–47. 3756460 10.1016/0093-934x(86)90032-5

[48] Weismer G, Martin RB. Acoustic and perceptual approaches to the study of intelligibility. Intelligibility in Speech Disorders: Theory, measurement and management. 1992 4 16;1:67.

[49] Ackermann H, Ziegler W. Articulatory deficits in parkinsonian dysarthria: an acoustic analysis. Journal of Neurology, Neurosurgery & Psychiatry. 1991 12 1;54(12):1093–8. 10.1136/jnnp.54.12.1093 PMC1014687 1783924

[50] Ziegler W, Wessel K. Speech timing in ataxic disorders: sentence production and rapid repetitive articulation. Neurology. Lippincott Williams & Wilkins; 1996 7;47(1):208–14. 8710079 10.1212/wnl.47.1.208

[51] Lindblom B. Explaining Phonetic Variation: A Sketch of the H&H Theory In: Speech Production and Speech Modelling. Dordrecht: Springer Netherlands; 1990 pp. 403–39.

[52] Liberman AM, Mattingly IG. The motor theory of speech perception revised. Cognition. Elsevier BV; 1985 10;21(1):1–36. 4075760 10.1016/0010-0277(85)90021-6

[53] Farnetari E, Recasens D. Coarticulation and Connected Speech Processes In: Hardcastle WJ, Laver J, Gibbon FE, editors. The Handbook of Phonetic Sciences. Oxford, UK: Blackwell Publishing Ltd; 1997 pp. 316–52.

[54] Baese-Berk M, Goldrick M. Mechanisms of interaction in speech production. Language and Cognitive Processes. Informa UK Limited; 2009 5;24(4):527–54. doi: 10.1080/01690960802299378 19946622 10.1080/01690960802299378 PMC2782840

[55] Scarborough R. Neighborhood-conditioned patterns in phonetic detail: Relating coarticulation and hyperarticulation. Journal of Phonetics. Elsevier BV; 2013 11;41(6):491–508.

[56] Mücke D, Grice M, Cho T. More than a magic moment–Paving the way for dynamics of articulation and prosodic structure. Journal of Phonetics. 2014 5 31;44:1–7.

[57] Nelson NR, Wedel A. The phonetic specificity of competition: Contrastive hyperarticulation of voice onset time in conversational English. Journal of Phonetics. Elsevier Ltd; 2017 9 1;64:51–70.

[58] Hall KC, Hume E, Jaeger F, Wedel A. The Message Shapes Phonology: A Unified Account of Strong and Weak Patterns. Stanford: Department of Linguistics. Language; under review.

[59] Singer C, Sanchez-Ramos J, Weiner WJ. Gait abnormality in essential tremor. Movement Disorders. Wiley-Blackwell; 2004 10;9(2):193–6. 10.1002/mds.870090212 8196682

[60] Klebe S, Stolze H, Grensing K, Volkmann J, Wenzelburger R, Deuschl G. Influence of alcohol on gait in patients with essential tremor. Neurology. 2005 7 11;65(1):96–101. doi: 10.1212/01.wnl.0000167550.97413.1f 16009892 10.1212/01.wnl.0000167550.97413.1f

[61] Arkadir D, Louis ED. The balance and gait disorder of essential tremor: what does this mean for patients? Therapeutic Advances in Neurological Disorders. SAGE Publications; 2013 1;6(4):229–36. doi: 10.1177/1756285612471415 23858326 10.1177/1756285612471415 PMC3707350

[62] Kronenbuerger M, Gerwig M, Brol B, Block F, Timmann D. Eyeblink conditioning is impaired in subjects with essential tremor. Brain. Oxford University Press (OUP); 2007 4;130(6):1538–51. 17468116 10.1093/brain/awm081

[63] Helmchen C, Hagenow A, Miesner J, Sprenger A, Rambold H, Wenzelburger R, et al Eye movement abnormalities in essential tremor may indicate cerebellar dysfunction. Brain. Oxford University Press (OUP); 2003 6;126(6):1319–32. 12764054 10.1093/brain/awg132

[64] Montgomery JEB. Deep Brain Stimulation Programming: Principles and Practice. Oxford University Press; 2010.

[65] Becker J, Barbe MT, Hartinger M, Dembek TA, Pochmann J, Wirths J, et al The Effect of Uni- and Bilateral Thalamic Deep Brain Stimulation on Speech in Patients With Essential Tremor: Acoustics and Intelligibility. Neuromodulation: Technology at the Neural Interface. Wiley-Blackwell; 2017 2;20(3):223–32. 10.1111/ner.12546 28160355

[66] Fasano A, Herzog J, Raethjen J, Rose FEM, Muthuraman M, Volkmann J, et al Gait ataxia in essential tremor is differentially modulated by thalamic stimulation. Brain. Oxford University Press (OUP); 2010 10;133(12):3635–48. 20926368 10.1093/brain/awq267

[67] Groppa S, Herzog J, Falk D, Riedel C, Deuschl G, Volkmann J. Physiological and anatomical decomposition of subthalamic neurostimulation effects in essential tremor. Brain. Oxford University Press (OUP); 2013 11;137(1):109–21. 24277721 10.1093/brain/awt304

[68] Darley FL, Aronsnon AE, Brown JR. Motor speech disorders, Saunders W. B, Philadelphia 1975:171–975.

[69] Klein JC, Lorenz B, Kang J-S, Baudrexel S, Seifried C, van de Loo S, et al Diffusion tensor imaging of white matter involvement in essential tremor. Hum Brain Mapp. Wiley-Blackwell; 2010 6;32(6):896–904. doi: 10.1002/hbm.21077 20572209 10.1002/hbm.21077 PMC6870356

