# Supplementary material for: The effects of Thalamic Deep Brain Stimulation on speech dynamics in patients with Essential Tremor: An articulographic study
Source: PLoS One. 2018 Jan 23;13(1):e0191359. doi: 10.1371/journal.pone.0191359 (PMC5779681; doi:10.1371/journal.pone.0191359)
Supplement: S1 Appendix — Stereotactic coordinates from 24 electrodes with reference to the midcommissural point (MCP). Stimulation parameters: amplitude (V; mA), pulse duration (μsec) and stimulation frequency (Hz). (PDF) [file pone.0191359.s001.pdf]

S 1 Appendix: Stereotactic coordinates

Nr.	*Sex*	*Age [years]*	*Years of Dis. Dur.*	*Months of DBS*	*x-coordinate [mm]*	*y-coordinate [mm]*	*z-coordinate [mm]*	*Stimulation parameters*
1	m	66	22	25	1: -11.8; 2: -12.5; 3: -13.3	1: -4.7; 2:-3.3; 3: -2.1	1: -1; 2: -0.1; 3: 0.9	L: case+, 1-; 1.4V; 2-; 3-; 1.7V; 60µs; 125Hz
					9: 12.1; 10: 12.3; 11: 12.8	9: -4.1; 10: -2.6; 11: -1.6	9: -0.1; 10: 1.4; 11: 2.9	R: case+; 9-; 1.6V; 10-; 11-; 2.4V; 60µs; 125Hz
2	m	70	29	5	1: -12.6	1: -4.2	1: -1	L: case+; 1-; 1mA; 60µs; 130Hz
					9: 12.7	9: -5.1	9: 0.4	R: case+; 9-; 1mA; 60µs; 130Hz
3	m	31	9	105	0: -9.9	0: -5.5	9: -1.2	L: case+; 0-; 3.5V; 60µs; 150Hz
					8: 9.3	8: -5.5	8: -3.5	R: case+; 8-; 3.8V; 60µs; 150Hz
4	m	73	34	56	0: -12.6; 1: -13.1	0: -5.8; 1: -4.6	0: -1.5; 1: -0.1	L: case+; 0-; 1-; 2V; 90µs; 150Hz
					10: 14	10: -4.6	10: 0	R: case+; 10-; 2.2V; 90µs; 150Hz
5	f	53	4	47	1: -10.6; 2: -11.3	1: -3.7; 2: -2	1: 1.2; 2: 2.4	L: case+; 1-; 3.5V; 2- 4V; 90µs; 125Hz
					9: 10.7; 10: 11.3	9: -3.2; 10: -1.6	9: 1.4; 10: 2.5	R: case+; 9-; 3.5V; 10-; 4V; 90µs; 125Hz
6	f	59	4	33	2: -11.1; 3: -11.5	2: -4; 3: -2.7	2: 1.1; 3: 2.3	L: case+; 2-; 2.4V; 3-; 1.8V; 90µs; 120Hz
					10: 12.2	10: -4.2	10: 1.7	R: case+; 10-; 2.8V; 60µs; 120Hz
7	m	54	7	67	1: -11.8; 2: -12.5	1: -3.7; 2: -1.7	1: 2.1; 2: 3.8	L: case+; 1-; 0.3V; 2-; 1.1V; 60µs; 120Hz
					9: 11.3; 10: 12	9: -3.2; 10: -1.7	9: 1.3; 10: 2.8	R: case+; 9-; 10-; 1.3V; 60µs; 120Hz
8	f	60	14	93	0: -10.7	0: -6.2	0: -1.2	L: case+; 0-; 1.5V; 60µs; 125Hz
					8: 8.8; 9: 9.3; 10: 10.5	8: -7.1; 9: -6; 10: -4	8: -2.1; 9: -0.8; 10: 1.1	R: case+; 8-; 1V; 9-; 10-; 1.4V; 60µs; 125Hz
9	f	61	1	4	2: -11.2	2: -4.8	2: 1.4	L: case+; 2-; 6.5mA; 60µs; 130Hz
					10: 10.5	10: -4.1	10: 1.8	R: case+; 10-; 4mA; 60µs; 130Hz
10	m	67	24	66	0: -9.2; 1: -10	0: -5.7; 1: -4.6	0: -0.7; 1: 0.9	L: case+; 0-; 3.1V; 1-; 1.3V; 60µs; 120Hz
					9: 11.9; 10: 12.8	9: -4.3; 10: -2.6	9: 0.6; 10: 1.8	R: case+; 9-; 2.1V; 10-; 2.8V; 60µs; 120Hz
11	m	72	9	7	1: -11.9	1: -5.7	1: -1	L: case+; 1-; 1.1mA; 60µs; 130Hz
					9: 12.4	9: -6.6	9: -0.8	R: case+; 9-; 1mA; 60µs; 130Hz
12	m	73	7	81	1: -11.8; 2: -12.5	1: -6.8; 2: -4.5	1: 0.9; 2: 2.2	L: case+; 1-; 2V; 2-; 2.3V; 60µs; 120Hz
					8: 11.8	8: -7.1	8: -1.1	R: case+; 8-; 0.5V; 60µs; 120Hz
**Mean (± SD)**		**61.6 (±11.9)**	**13.7 (±10.9)**	**49.1 (±34.8)**				
